# Risk for Gestational Diabetes Mellitus and Adverse Birth Outcomes in Chinese Women with Polycystic Ovary Syndrome

**DOI:** 10.1155/2016/5787104

**Published:** 2016-03-14

**Authors:** Qing Xiao, Yong-Yi Cui, Jine Lu, Guo-Zheng Zhang, Fang-Ling Zeng

**Affiliations:** Department of Obstetrics and Gynecology, Guangzhou Women and Children's Medical Center, Guangzhou Medical University, Guangzhou 510623, China

## Abstract

*Objective.* To examine the association of polycystic ovary syndrome (PCOS) in early pregnancy with gestational diabetes mellitus (GDM) and adverse birth outcomes.* Methods.* In this retrospective cohort study including 2389 pregnant women, the medical records of 352 women diagnosed with PCOS were evaluated. Outcomes included GDM, preterm birth, low birth weight, macrosomia, and being small and large for gestational age. Multivariable logistic regression models were used to examine the association of the risk for GDM and adverse birth outcomes with PCOS after adjusting for confounders.* Results.* Women previously diagnosed with PCOS had a higher risk of GDM (adjusted odds ratio [OR] 1.55, 95% confidence interval [CI]: 1.14–2.09). A strong association was seen between PCOS and preterm birth (adjusted OR 1.69, 95% CI: 1.08–2.67). On stratified analysis, the adjusted OR for GDM among women with PCOS undergoing assisted reproductive technology was 1.44 (95% CI: 1.03–1.92) and among women with PCOS who conceived spontaneously was 1.60 (1.18–2.15). No increased risk for other adverse birth outcomes was observed.* Conclusions.* Women with PCOS were more likely to experience GDM and preterm birth.

## 1. Introduction

Polycystic ovary syndrome (PCOS), one of the most common endocrine disorders occurring during reproductive age, is characterized by ovulatory dysfunction, biochemical or clinical hyperandrogenism, and polycystic ovaries [1]. Its prevalence ranges from 5% to 20% depending on the diagnostic criteria used [2, 3]. PCOS is currently considered a syndrome with metabolic consequences that could affect women's health during different stages of reproductive age [4].

Several studies have highlighted that the risk for maternal, neonatal, and obstetric complications may be increased in women with PCOS [5–7]. Gestational diabetes mellitus (GDM) is the most commonly reported pregnancy complication in women with PCOS. Pregnant women with PCOS have been reported to develop insulin resistance and impaired *β*-cell function [8]. This pathogenic mechanism may be associated with glucose intolerance, resulting in a greater incidence of GDM in women with PCOS. Observational studies have revealed an association between PCOS and GDM, hypertension during pregnancy, and preterm birth [2]. However, these studies are limited by significant heterogeneity, which indicates that the reliability of the finding of increased risk of pregnancy and adverse birth outcomes in women with PCOS could be compromised [9, 10]. Therefore, properly designed studies should be performed before formulating recommendations for pregnant women with PCOS. Determining the risk for GDM and adverse birth outcomes in women with PCOS is important for preventive intervention through screening in the early stage of pregnancy.

We conducted a large historic cohort study of pregnant women, including those who conceived spontaneously and through assisted reproductive technology, to assess the risk for GDM and adverse birth outcomes among Chinese women with PCOS.

## 2. Materials and Methods

### 2.1. Participants

This historic cohort study was performed at Guangzhou Women and Children's Medical Center (GWCMC), China, between January 1, 2011, and December 31, 2014. The inclusion criteria were singleton pregnancies, <13 weeks of gestation at the first antenatal visit, and history of screening for GDM. Exclusion criteria were multiple gestation pregnancies, history of preexisting diabetes, and missing delivery information. In total, data on 2389 deliveries were obtained from electronic medical records, including demographic data, maternal medical history, and labor and delivery information. We examined outpatient medical records to identify women who were diagnosed at least once with PCOS before early pregnancy (<13 weeks of gestation). PCOS was diagnosed according to the Rotterdam 2003 criteria [11], with presence of at least two of three criteria, including polycystic ovaries, oligomenorrhea, and hyperandrogenism. Polycystic ovaries were detected by ultrasound and defined as 12 or more follicles of 2–9 mm and ovarian volume ≥10 mL in at least one ovary. Oligomenorrhea was defined by a length of menstrual cycle >35 days or <10 periods/year. Hyperandrogenism was defined based on laboratory and/or clinical symptoms. The only assisted reproductive technology women underwent was in vitro fertilization.

### 2.2. GDM and Birth Outcomes

#### 2.2.1. Diagnosis of GDM

All pregnant women at the antenatal clinic in GWCMC underwent a routine 75 g oral glucose tolerance test between 24 and 28 weeks of gestation. GDM was diagnosed according to the modified International Association of Diabetes and Pregnancy Study Groups (IADPSG) criteria when one or more of the following glucose levels were elevated: fasting plasma glucose level ≥5.1 mmol/L, 1 h plasma glucose level ≥10.0 mmol/L, and 2 h plasma glucose level ≥8.5 mmol/L.

#### 2.2.2. Preterm Birth

Preterm birth was defined as birth at <37 weeks of gestation, classified as moderately (32^+0^ to 36^+6^ weeks) and very preterm birth (<32 weeks).

#### 2.2.3. Small (SGA) and Large (LGA) for Gestational Age

SGA was defined by a fetal growth less than the 10th percentile at each completed week of gestation, and LGA was defined by a fetal growth greater than the 90th percentile, according to the report on Chinese infants born between 28 and 44 weeks of gestation in 2014 [12]. We defined SGA and LGA at less than 28 weeks of gestation based on the United States national reference [13], because of the lack of a reference for the Chinese population.

#### 2.2.4. Low Birth Weight and Macrosomia

Low birth weight (LBW) was defined as a birth weight <2500 g. Macrosomia was defined as a birth weight ≥4000 g.

#### 2.2.5. Potential Confounders

Potential confounders included characteristics with a possible association with GDM, preterm birth, and fetal growth, including maternal age, maternal education, parity, prepregnancy body mass index (BMI), use of assisted reproductive technology, gestational age at delivery, and newborn sex. Data were obtained from hospital medical records.

### 2.3. Ethics Statement

The institutional review board of Guangzhou Women and Children's Medical Center approved the study.

### 2.4. Statistical Analysis

We compared women with PCOS and those without PCOS group using the chi-squared test for categorical variables and *t*-test for continuous variables. Multivariable logistic regression models were used to examine the association of the risk for GDM and adverse birth outcomes with PCOS after adjusting for confounders. The crude and adjusted odds ratios (ORs) with 95% confidence intervals (95% CIs) were computed to estimate the degree of association.

All *P* values <0.05 were considered statistically significant. Statistical analysis was performed using the statistical package SPSS, version 20 (SPSS Inc., Chicago, IL).

## 3. Results

Demographic characteristics of mothers and newborns according to PCOS status are presented in Table 1. A total of 352 women reported a history of PCOS. Women with PCOS before early pregnancy were more likely to be older, had higher prepregnancy BMI, and used assisted reproductive technology compared with women without PCOS.

Table 2 presents multivariate associations of PCOS during pregnancy with GDM and birth outcomes. In the adjusted analysis, women with a previous diagnosis of PCOS had a higher risk for GDM than women with no such diagnosis (adjusted OR 1.55, 95% CI: 1.14–2.09). There was also a strong association between PCOS and preterm birth (adjusted OR 1.69, 95% CI: 1.08–2.67).

**Figure 1:**
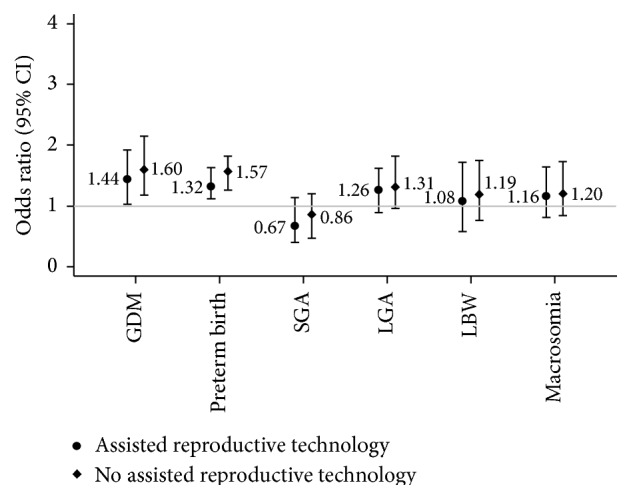
Odds ratios of gestational diabetes mellitus (GDM) and adverse birth outcomes in women with and without polycystic ovary syndrome (PCOS) undergoing assisted reproductive technology (adjusted for maternal age at birth, parity, education, and prepregnancy body mass index).

In the stratified analysis using multivariable logistic regression, the adjusted OR for GDM among women with PCOS undergoing assisted reproductive technology was 1.44 (95% CI: 1.03–1.92) and among women with PCOS who conceived spontaneously was 1.60 (1.18–2.15) (Figure 1). Also, the risk of preterm birth was increased in women with PCOS regardless of use of assisted reproductive technology. There was no difference in the incidence of other adverse birth outcomes.

## 4. Discussion

The present study, in which maternal age at birth, parity, education, prepregnancy body mass index, and use of assisted reproductive technology were controlled for, indicated that a previous diagnosis of PCOS increases the risk of GDM and preterm birth, although no increased risk for other adverse birth outcomes was observed.

A 1.5-fold increased risk for GDM in women with PCOS in early pregnancy was seen in our study. This finding is consistent with that of another study, which reported the incidence of GDM to be 19.2% in Jewish women, 44.4% in Iranian women, 40.9% in American women, and 26.9% in Mexican women with PCOS compared with 3%, 7.3%, 9.4%, and 9.6% in women without PCOS, respectively [3, 14–16]. These findings show that PCOS has a different impact on the risk of GDM depending on ethnicity. There are three possible explanations: (1) PCOS has different characteristics and clinical impact in different ethnic groups, (2) increased insulin resistance in PCOS has different clinical effects depending on the insulin metabolism characteristic of different ethnicities and environments, and (3) the prevalence of GDM in women with PCOS is affected by the different diagnosis criteria (the WHO or the modified IADPSG criteria) [3, 17]. In addition, PCOS is a major cause of infertility in women, and these women might require assisted reproductive technology to become pregnant [18]. Some studies have suggested that assisted reproductive technology is associated with an increased risk of GDM [19–21], which indicated that women with pregnancies that were conceived while undergoing assisted reproductive technology have impaired glucose tolerance compared with those who conceived spontaneously. Of note, most prior studies have not examined PCOS status independently of assisted reproductive technology. In this setting, we did a stratified analysis in two separate groups, one restricted to women undergoing assisted reproductive technology versus those who were not. After regression analysis of the two separate groups, we showed that women with PCOS had a greater risk of GDM during pregnancy regardless of assisted reproductive technology. Women with PCOS were associated with an increased risk of GDM that could not be attributed to the increased use of assisted reproductive technology. This may explain our findings that PCOS, an inherent insulin resistant condition, is independently associated with GDM.

Our study showed that women with PCOS had an increased risk of preterm birth. Similarly, a large population based cohort study found that PCOS was strongly associated with very preterm birth [2], and a systematic review showed that PCOS increased the risk of preterm birth by at least 2-fold [22]. However, the pathophysiological mechanisms underlying the association between PCOS and preterm birth are not completely understood. Various etiologies have been suggested, including the increased incidence of multiple pregnancies and nulliparity [23], the associated increased estrone levels, hyperinsulinemia, and the subsequent diabetic and hypertensive predispositions [5, 24, 25]. In previous reports, women with PCOS often required assisted reproductive technology to become pregnant, increasing the risk of multiple births and hypertensive disease, which are associated with preterm birth [26–28]. The association between PCOS and preterm birth may thus be an interaction with assisted reproductive technology. In our stratified analysis, the results did not support the statement that adverse pregnancy outcomes among women with PCOS were mediated by assisted reproductive technology. There was no significant association between the interaction of PCOS with assisted reproductive technology and preterm birth. This indicates that PCOS is also an independent risk factor of preterm birth. This finding is supported by two studies from Northern Europe [2, 29], which reported that preterm birth associated with assisted reproductive technology could be explained by factors that lead to infertility, rather than the assisted reproductive technology. In the current study, we could not identify the pathophysiological mechanisms behind the increased risk of preterm birth among women with PCOS, a potential relationship that should be addressed in future studies.

A major strength of this study was the relatively large sample size and the fact that potential confounding variables that were not controlled for in most previous studies were controlled for in this study. We also included women who conceived spontaneously and those who conceived by assisted reproductive technology to study pregnancy outcomes. However, certain limitations must be noted. The sample size was based on the primary outcome and is therefore not suitable for estimating the risk for all perinatal outcomes such as stillbirth and neonatal death. Because the study was a retrospective cohort study, hormone levels were not measured to examine individual PCOS status, and data on medical therapy for PCOS were not available.

In summary, our results suggest that women with PCOS were more likely to develop GDM and experience preterm birth. Future longitudinal studies are needed to better determine the underlying processes of PCOS during gestation and to develop efficient preventive strategies to preclude the adverse effects on both the mother and child.

## Figures and Tables

**Table 1:** Maternal and newborn characteristics according to polycystic ovary syndrome (PCOS) status.

Characteristics	Women with PCOS	Women without PCOS	*P* value
	(*n* = 352)	(*n* = 2037)	
Maternal characteristics			
Age at birth (years)	29.7 ± 3.6	28.6 ± 3.2	<0.001
<25	26 (7.4)	170 (8.3)	<0.001
25–29	158 (44.9)	1180 (57.9)	
30–34	137 (38.9)	584 (28.7)	
≥35	31 (8.8)	103 (5.1)	
Education			
Junior high school or lower	38 (11.1)	191 (9.8)	0.628
High school	100 (29.2)	559 (28.7)	
College	161 (47.1)	982 (50.4)	
Undergraduate or higher	43 (12.6)	216 (11.1)	
Missing	10	89	
Prepregnancy BMI			
≤18.4	35 (9.8)	228 (11.2)	<0.001
18.5–24.9	269 (76.4)	1695 (83.2)	
25.0–29.9	43 (12.3)	102 (5.0)	
≥30.0	5 (1.5)	12 (0.6)	
Parity			
0	338 (96.0)	1855 (91.1)	0.002
≥1	14 (4.0)	182 (8.9)	
Assisted reproductive technology			
Yes	49 (13.9)	52 (2.6)	<0.001
No	303 (86.1)	1985 (97.4)	
Newborn characteristics			
Sex			
Male	187 (53.1)	1092 (53.6)	0.867
Female	165 (46.5)	945 (46.4)	
Mean gestational age (weeks)	39.1 ± 1.2	39.3 ± 1.1	0.103
Mean birth weight (g)	3253 ± 422	3223 ± 406	0.194
Delivery mode			
Vaginal delivery	224 (63.6)	1322 (64.9)	0.647
Cesarean delivery	128 (36.4)	715 (35.1)	

BMI, body mass index.

Data are expressed as mean ± standard deviation or *n* (%).

**Table 2:** Risk of gestational diabetes mellitus (GDM) and adverse birth outcomes in women with polycystic ovary syndrome (PCOS) versus women without PCOS.

Characteristics	Women with PCOS (*n* = 352)	Women without PCOS (*n* = 2037)	Crude odds ratio (95% CI)	Adjusted odds ratio (95% CI)^*∗*^
GDM				
Yes	64 (18.2)	278 (13.6)	1.52 (1.13, 2.03)	1.55 (1.14, 2.09)
No	288 (81.8)	1759 (86.4)	Reference	Reference
Preterm birth				
Yes	30 (8.5)	94 (4.6)	1.93 (1.26, 2.95)	1.69 (1.08, 2.67)
No	322 (91.5)	1943 (95.4)	Reference	Reference
Small for gestational age				
Yes	31 (8.8)	194 (9.5)	0.92 (0.62, 1.37)	0.79 (0.51, 1.23)
No	321 (91.2)	1843 (90.5)	Reference	Reference
Large for gestational age				
Yes	43 (12.2)	181 (8.9)	1.43 (1.00, 2.03)	1.39 (0.98, 1.99)
No	309 (87.8)	1856 (91.1)	Reference	Reference
Low birth weight				
Yes	21 (6.0)	108 (5.3)	1.13 (0.70, 1.84)	1.20 (0.74, 1.95)
No	331 (94.0)	1929 (94.7)	Reference	Reference
Macrosomia				
Yes	23 (6.5)	87 (4.3)	1.56 (0.98, 2.52)	1.21 (0.94, 2.01)
No	329 (93.5)	1950 (95.7)	Reference	Reference

^*∗*^Adjusted for maternal age at birth, parity, education, prepregnancy body mass index, and assisted reproductive technology.
